# Ciliated hepatic foregut cyst: a report of 6 cases and a review of the English literature

**DOI:** 10.1186/s13000-015-0321-1

**Published:** 2015-06-30

**Authors:** Katherine C. Bishop, Carmen M. Perrino, Marianna B. Ruzinova, Elizabeth M. Brunt

**Affiliations:** Washington University School of Medicine, 660 South Euclid Avenue, Saint Louis, MO 63110 USA; Department of Obstetrics and Gynecology, Duke University School of Medicine, 40 Duke Medicine Cir #1J, Durham, NC 27710 USA; Department of Pathology and Immunology, Washington University School of Medicine, Campus Box 8118, 660 South Euclid Avenue, Saint Louis, MO 63110 USA

**Keywords:** Ciliated hepatic foregut cyst, Liver, Cyst

## Abstract

**Background:**

Ciliated hepatic foregut cyst (CHFC) is a rare cystic lesion most commonly identified in segment 4 of the liver that arises from the embryonic foregut. The classic histologic pattern is comprised of 4 distinct layers (inner ciliated epithelial lining, smooth muscle, loose connective tissue, fibrous capsule). Although rare, cases of metaplastic and malignant epithelial lining have been described in CHFC.

**Methods:**

We report 6 additional cases of CHFC, one of which had gastric metaplasia of the cyst lining, and review all reported cases of CHFC in the English literature. We describe the clinicopathologic analysis of 6 cases, with selective immunohistochemical analysis on 1 case with gastric metaplasia.

**Results:**

Cases occurred in 4 women and 2 men (average age 55 years, range 42 to 67 years). Cysts ranged in size from 0.7 to 17 cm (average 7.2 cm) and were grossly tan-pink to white with blood-filled contents. The majority were located in segment 4 of the liver, however 2 were located in the porta hepatis. Tumor serologies (CA19-9 and/or CEA) were performed in 3 cases; 1 case demonstrated elevated CA19-9, and 2 cases had laboratory values within normal limits. All cases showed the classic histologic findings, however one case additionally had extensive gastric metaplasia.

**Conclusions:**

In conclusion, CHFC is a rare diagnostic entity that should be considered in the differential diagnosis for cystic hepatic lesions, particularly those located in segment 4 of the liver. Metaplasia and squamous carcinoma can occur, therefore complete surgical excision is the recommended treatment.

**Electronic supplementary material:**

The online version of this article (doi:10.1186/s13000-015-0321-1) contains supplementary material, which is available to authorized users.

## Background

Ciliated hepatic foregut cysts (CHFC) are rare cystic lesions of embryological origin that have been increasingly diagnosed in the last few decades [1]. Wheeler and Edmondson first coined this term and described its defining characteristics in 1984 [2]. CHFCs typically consist of four layers: (1) an inner layer of ciliated, pseudostratified columnar epithelium; (2) loose lamina propria; (3) a smooth muscle band, ranging from one to three layers in thickness; and (4) an outer fibrous capsule [3]. The cysts are often small (measuring less than 4 cm), unilocular, subcapsular, and located in segment 4 of the left lobe of the liver [3, 4]. They are frequently discovered incidentally and are usually benign. While no reports exist of recurrence following complete surgical excision, CHFC has been reported to enlarge over time following biopsy and may recur after sclerotherapy [5–8].

The esophagus and trachea form from the dorsal foregut, while the liver forms from the ventral foregut; the similar features of CHFC compared to bronchial and esophageal cysts are presumably due to this common derivation of the liver, esophagus, and tracheobronchial tree from the embryological foregut [3]. There are no other known entities that are primary to the liver and have ciliated epithelium, thus distinguishing CHFC from other hepatic cysts [2].

Over one hundred cases of CHFC have been reported to date in the world literature [4]. Among these cases, six developed squamous metaplasia without evidence of dysplasia or malignancy and five developed squamous cell carcinoma of the innermost cyst lining [7, 9–19]. One case showed both evidence of gastric antral metaplasia and squamous cell carcinoma [16]. While metaplasia within a CHFC is a rare occurrence, its significance must not be overlooked given the subsequent risk of malignant transformation.

Six cases of CHFC have been identified at our institution over a 24 year time period. One of these cases showed extensive gastric metaplasia. In this report we aim to describe these six cases, as well as to provide a detailed review of the English literature regarding this rare diagnostic entity with a focus on metaplasia of the ciliated epithelial lining.

## Case Presentation

### Methods

 A search of the pathology database at our institution from 1989 to 2013 identified six cases of CHFC. Hematoxylin and eosin-stained slides and immunohistochemical stains (if performed) were reviewed for each case. The patient demographics, clinical presentation, imaging studies, and follow-up were obtained through available clinical records. This study received approval from the Human Resources and Protection (HRPO) Office and Institutional Review Board (IRB) Committee at Washington University.

### Clinical summary

Clinicopathologic characteristics of the patients are summarized in Table 1. Four of the six patients were women and two were men, with ages ranging from 42 to 67 years (average 55 years). Only two patients were symptomatic at presentation, each initially presenting with right upper quadrant pain. One of these patients (Case 4) also developed jaundice due to biliary obstruction caused by the cyst; his laboratory values included elevated lipase (4540 Units/L, normal range 0–99 Units/L), amylase (1149 Units/L, normal range 28–100 Units/L), bilirubin (20.1 mg/dL, normal range 0.3-1.1 mg/dL), alkaline phosphatase (530 Units/L, normal range 38–126 Units/L), aspartate transaminase (62 Units/L, normal range 11–47 Units/L), alanine transaminase (109 Units/L, normal range 7–53 Units/L), and CA19-9 (398.6 Units/mL, normal range 0–36 Units/mL), and CEA within normal limits (1.0 ng/mL, normal range 0–2.5 ng/mL). Two additional patients had tumor marker serologies (Case 1, CA19-9; Case 6, CA19-9 and CEA) within normal limits, and the remaining three (Cases 2, 3, 5) did not have these laboratory tests performed. Two patients had colorectal cancer, and their cysts were discovered incidentally during surgery. Five of the six cysts were surgically resected, one of which was only partially resected because the patient declined to undergo an additional procedure for complete enucleation. One patient underwent core biopsy followed by observation. One patient was presumed to have a mucinous cystic neoplasm of the pancreas after undergoing imaging studies and ultrasound-guided fine needle aspiration (FNA) biopsy performed at an outside institution (Case 6). Despite repeat imaging at our institution, it was only after the patient was taken to the operating room that the lesion was identified as originating from the porta hepatis of the liver, not the pancreas.

**Table 1:** Six additional cases of ciliated hepatic foregut cysts

Case	Age (yrs)/ sex	Clinical presentation	Tumor markers	Size (cm)	Location	Locularity	Treatment	Metaplasic epithelium	Cyto/Bx	Follow-up	Pertinent history
1	42/F	PP epigastric discomfort	WNL	8 x 4 x 4	Porta hepatis	Uni	Resection	Gastric	N	LTFU	---
2	58/F	Incidental	None	0.7 x 0.6 x 0.5	S4b	Uni	Resection	N	N	LTFU	CRC with liver mets
3	46/F	Incidental	None	1 x 0.5 x 0.3	Right liver	Uni	Observation	N	Bx	Alive	CRC
4	66/M	RUQ pain, N/V, J, WL	↑CA19-9	17 x 16 x 15	S4	Uni	Partial resection	N	N	Alive	---
5	50/F	Incidental	None	10 x 5.5 x 2	NR	^a^Uni	Resection	N	N	LTFU	---
6	67/M	Incidental	WNL	6.5 x 4.0 x 2.0	Porta hepatis	Uni	Resection	N	Cyto	Alive	Presumed pancreatic head mass

^a^Case 5, gross description describes cyst wall comprised of “irregular fibrous trabeculae”

*Bx* biopsy; *Cm* centimeters; *CRC* concomitant colorectal cancer; *Cyto* cytology; *F* female; *J* jaundice; *LTFU* lost to follow-up; *M* male; *Mets* metastases; *N* no; *NR* not reported; *N/V* nausea/vomiting; *PP* post-prandial; *RUQ* right upper quadrant; *S* segment (of liver); *WL* weight loss; *WNL* within normal limits; *Uni* unilocular; *Yrs* years

### Gross pathology

Grossly, the cysts were tan-pink in color, with one cyst having green-brown plaques on the inner surface. They contained white to bloody contents; specifically, one contained thick, tan, mucoid material, one contained white material, and one had a small amount of attached thrombus. The cysts ranged in size from 0.7 to 17 cm in greatest dimension (average 7.2 cm). All six cysts were unilocular. Five cysts had smooth walls, and one had numerous “irregular fibrous trabeculae” in the wall.

### Microscopic pathology

**Fig. 1:**
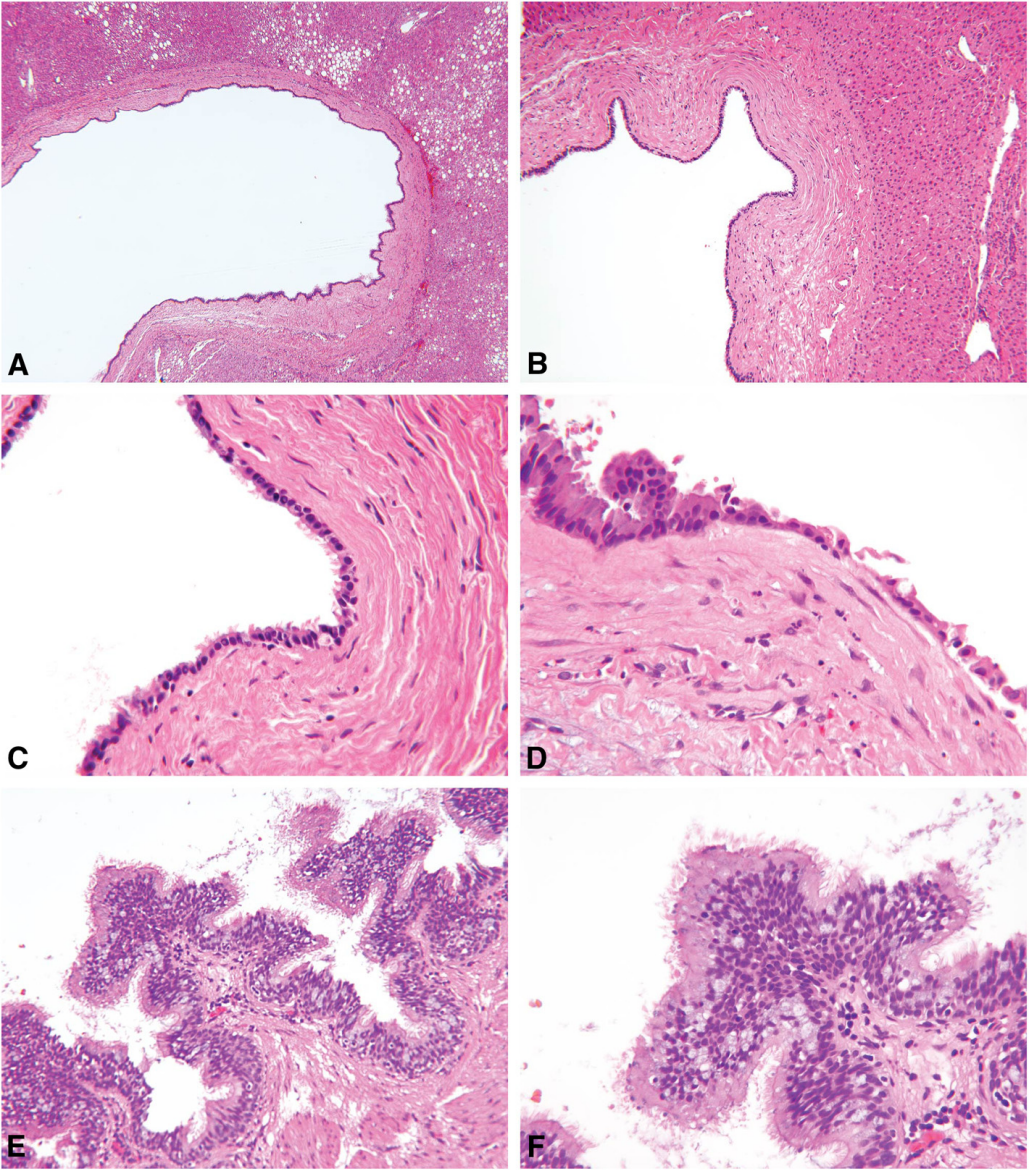
CHFC, classic cases **a** Case 2, low power view highlights classic cyst architecture. **b** Case 3, CHFC is surrounded by a fibrous capsule and well-demarcated from the surrounding hepatic parenchyma. **c** Case 3, innermost layer may be completely or incompletely lined by cilia. **d** Case 4, rare goblet cells (black circle) mimicking respiratory tract mucosa may be seen. **e** Case 6, ciliated, pseudostratified epithelium. **f** Case 6, high power view shows pseudostratified, ciliated epithelium with abundant goblet cells (all images hematoxylin-eosin; original magnifications X40 [A], X100 [B], X200 [E], X400 [C, D, F])

**Fig. 2:**
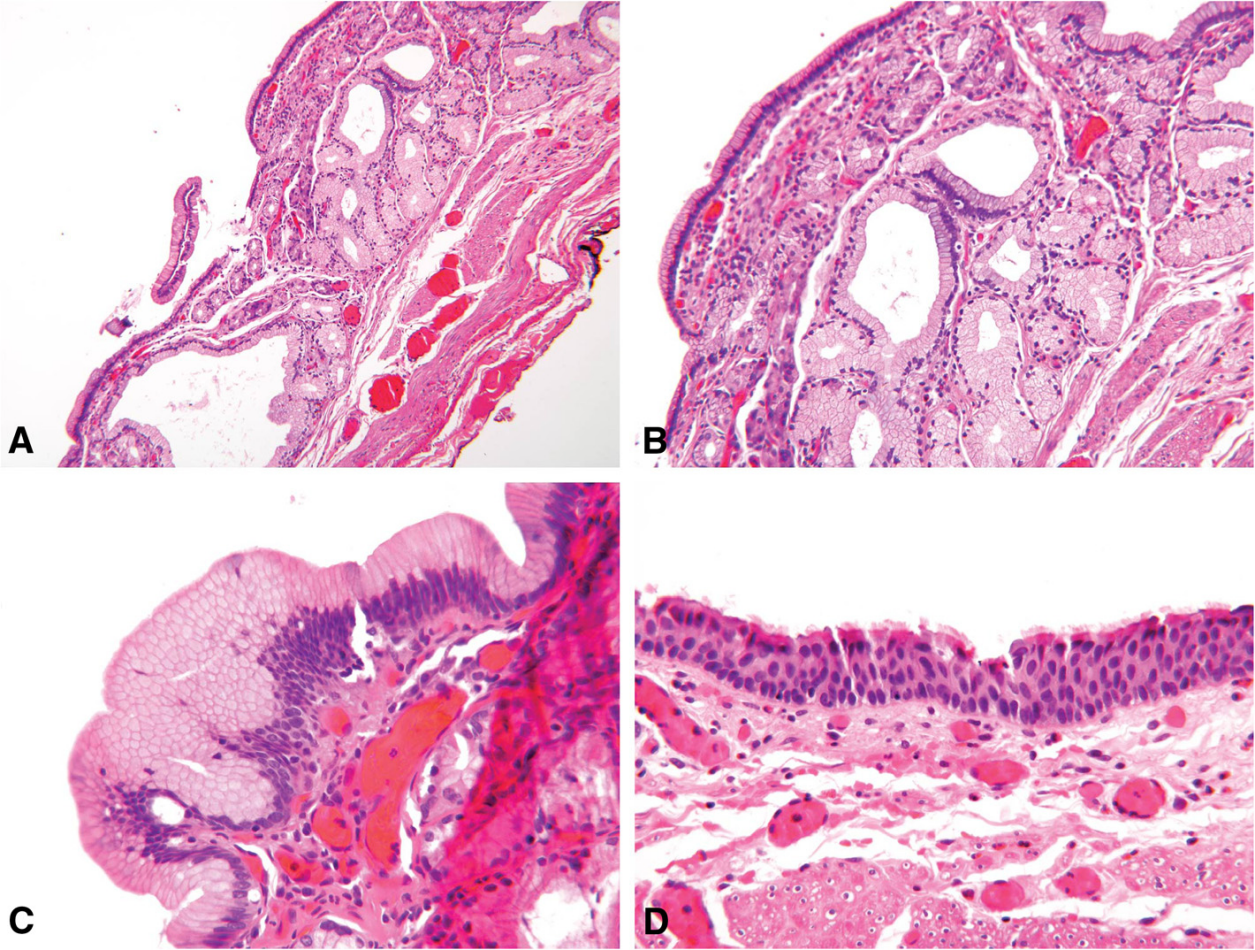
CHFC with gastric metaplasia **a** Case 4, CHFC with gastric-type mucus-secreting glands in the submucosa. **b** Case 4, CHFC with branching mucous glands. **c** Case 4, surface foveolar-type epithelium. **d** Case 4, classic ciliated, pseudostratified, epithelial cyst lining (all images hematoxylin-eosin; original magnifications X100 [A], X200 [B], X400 [C, D])

Histologic examination showed that five of the six cysts were entirely lined with simple, ciliated, cuboidal to columnar epithelium, surrounded by loose connective tissue, smooth muscle, and a poorly defined fibrous pseudocapsule (Fig. 1). Goblet cells containing mucin were identified in two of these cases (Figs. 1d-1f). Two cases showed marked pseudostratification of the ciliated epithelial lining (Figs. 1e-1f, 2d). In addition to focal areas with the classic epithelial lining, one case also showed areas with gastric metaplasia comprised of mucous glands and foveolar epithelium (Figs. 2a-2c). Immunohistochemistry showed the epithelial lining in this case was positive for keratin 7 and negative for CDX-2.

## Conclusions

CHFC are increasingly reported hepatic lesions thought to be derived from the embryonic foregut that most frequently are identified in segment 4 of the liver, although a minority occur in the right lobe [4, 8]. Over one hundred cases have been reported to date in the world literature, with bimodal reporting peaking in the late nineteenth century attributed to the popularity of autopsy and again in the late twentieth century with the increasing utilization and accuracy of radiologic studies [8]. A high number of cases have been reported in Japan; it is unclear whether this is due to increased awareness or increased prevalence of this entity [8]. Radiologic findings associated with CHFC include a hypoechoic appearance on ultrasound and a hyperdense appearance on computed tomography (CT) scan [4].

The differential diagnosis for these lesions includes biliary cyst, simple cyst, parasitic cyst, mucinous cystic neoplasm (biliary cystadenoma), and various cystic metastases, such as cystic neuroendocrine tumor or necrotic metastases. Rarely, endometrial cysts may present as cystic lesions in women. In a child, an epidermoid cyst or a mesenchymal hamartoma are common possibilities [7]. When identified in the gallbladder fossa, a pancreatic pseudocyst, choledochal cyst, or gallbladder duplication should also be considered [20].

The classic histologic appearance of these lesions consists of 4 layers: a ciliated inner epithelial lining, loose connective tissue, smooth muscle, and a fibrous capsule [3]. Electron microscopy has confirmed the presence of goblet cells containing mucous vesicles and cilia lining the cysts composed of doublets of microtubules arranged in a 9 + 2 pattern [8, 21]. While some have suggested treatment with injection of sclerosing agents, surgical resection is generally the preferred method of treatment given the potential, albeit rare, for malignant transformation [4, 22, 23]. Interestingly, all of the malignant cases reported have shown squamous differentiation.

A review of the English literature showed 83 cases published to date, which are summarized in Additional file 1 [2, 4–59]. Patients ranged from 20 weeks gestational age to 82 years with a slight female predominance (41 female, 38 male, 4 not reported). The lesions were most commonly discovered incidentally. Tumor marker serologies (CA19-9, CEA, and/or AFP) were performed in 17 cases and were elevated in 6 of (35.3 %, 6/17). All of the cases with elevated tumor marker serologies had benign histologic features and no evidence of metaplasia. The cysts were most commonly unilocular, with sizes ranging from 0.6 cm to 19.3 cm (average 4.5 cm). Patients were most frequently treated with surgical resection. Core biopsy was performed in 6 cases (4/6, 66.7 % diagnostic) and FNA biopsy was performed in 12 cases (9/12, 75 % diagnostic).

Twelve cases (14.5 %, 12/83) had histologic evidence of metaplastic epithelium lining the inner cyst wall [7, 9–19, 33]. Eleven of these cases had squamous metaplasia, 5 of which also reported squamous cell carcinoma, presumably due to malignant transformation of the metaplastic squamous epithelium [6, 8–11, 13–18]. One case had concomitant squamous cell carcinoma as well as gastric antral metaplasia, and one case had only gastric metaplasia [16, 33]. Tumor marker serologies were performed in 4 of the metaplastic/malignant cases (3 squamous cell carcinoma, 1 squamous metaplasia), and all were within normal limits [10, 13, 15, 17]. Our series reports the third case of gastric metaplasia arising within a CHFC, and it is the second known case of gastric metaplasia in CHFC not associated with malignancy.

In conclusion, CHFC is a rare entity with 89 cases reported to date in the English literature, including the 6 cases from the current study. All cases from this series demonstrated the classical histologic features, with the cyst wall comprised of 4 distinct layers. While the inner epithelial lining is most commonly ciliated and pseudostratified, rare cases with squamous metaplasia, squamous carcinoma, and/or gastric metaplasia have been reported. Interestingly, tumor serologies were elevated in a minority of benign cases and showed no correlation with metaplasia or malignant transformation. This rare diagnostic entity should be included in the differential diagnosis of cystic liver lesions, and complete surgical excision should be recommended given the potential for metaplastic or malignant squamous carcinoma and the inability to monitor disease progression.

### Consent

A waiver of informed consent was granted from the HRPO Office and IRB Committee at Washington University due to the fact that this is a retrospective case series and no changes were made to patient management as a result of this study. The Washington University HRPO/IRB Committee is willing to provide written documentation confirming their decision for review by the Editor-in-Chief of this journal.

## Additional file

Additional file 1:
**Review of ciliated hepatic foregut cysts previously reported in the English literature.** Table containing all cases of ciliated hepatic foregut cyst published to date in the English literature. Data includes patient demographics, clinical features, location, treatment, metaplasia, if biopsy or fine needle aspiration was performed, and tumor serologic markers.

## Abbreviations

CHFC: Ciliated hepatic foregut cyst
CT: Computed tomography
HRPO: Human resources and protection office
IRB: Institutional review board
FNA: Fine needle aspiration
