# Supplementary material for: Ciliated hepatic foregut cyst: a report of 6 cases and a review of the English literature
Source: Diagn Pathol. 2015 Jun 30;10:81. doi: 10.1186/s13000-015-0321-1 (PMC4486693; doi:10.1186/s13000-015-0321-1)
Supplement: Additional file 1: — Review of ciliated hepatic foregut cysts previously reported in the English literature. Table containing all cases of ciliated hepatic foregut cyst published to date in the English literature. Data includes patient demographics, clinical features, location, treatment, metaplasia, if biopsy or fine needle aspiration was performed, and tumor serologic markers. [file 13000_2015_321_MOESM1_ESM.docx]

**Additional file 1 Review of ciliated hepatic foregut cysts previously reported in the English literature**

|  | **n** | **Age/sex** | **Clinical presentation** | **Size (cm)** | **Location** | **Locularity** | **Treatment** | **Metaplastic epithelium** | **Cyto/Bx** | **Other** | **Tumor markers^A^** |
| --- | --- | --- | --- | --- | --- | --- | --- | --- | --- | --- | --- |
| **Khoddami 2013** | 2 | 3.5 yr/M | AP | 2.3 | S4 | Uni | Resection | N | N | Prenatal Dx (9 mo) | NR |
|  |  | 3.5 yr/M | Incidental | 3.5 | R lobe | Uni | Resection | Squamous | N | Mimicked hydatid cyst | NR |
| **Wilson 2013** | 1 | 34 yr/M | Incidental | 14 | S4, 5, 8 | NR | TACE, PVE, Resection | SCC | ^B^Bx | --- | WNL |
| **Ambe 2012** | 1 | 42 yr/M | AP | 7 | S4 | Uni | Resection | N | N | Contents with *Morganella morganni* | NR |
| **Fernández-Aceñero 2012** | 2 | 35/F | AP, dizziness | 7 | S4, 5 | Bi | Resection | N | N | --- | NR |
|  |  | 33/M | Incidental | 6.5 | S4 | Bi | Resection | N | N | --- | NR |
| **Yang 2012** | 1 | 65 yr/M | Incidental | 1.5 | S4 | Uni | Resection | N | N | --- | NR |
| **Fujita 2011** | 4 | 11 mo/F | AM | 19.3 | NR | Multi | Resection | N | N | --- | NR |
|  |  | 17 yr/F | Incidental | 6 | NR | Multi | Resection | N | N | --- | NR |
|  |  | 17 yr/F | AP | 6.4 | NR | Uni | Resection | N | N | --- | NR |
|  |  | 5 mo/M | AP, AD | 9.5 | NR | Multi | Resection | N | N | --- | NR |
| **Shimokawa 2011** | 1 | 41 yr/F | AP, AD, Fa | 7 | S4b | Multi | Resection | N | N | Mimicked hydatid cyst | NR |
| **Guérin 2010** | 4 | 11 mo/M | Incidental | 8.8 | S4, 5, 8 | Bi | Resection | N | N | Prenatal Dx (22 wks) | ↑AFP |
|  |  | 14 mo/F | Incidental | 5 | S5 | Bi | Resection | Squamous | N | Prenatal Dx (22 wks) | NR |
|  |  | 10 yr/M | Incidental | 2 | S3 | Bi | Resection | N | N | --- | NR |
|  |  | 10 yr/F | Incidental | 0.6 | S4 | Uni | Organ harvest | N | N | --- | NR |
| **Zaydfudim 2010** | 1 | 17 yr/F | AP, N/V | 6.5 | S4, 8 | Uni | Resection | N | ^B^US-Bx | --- | WNL |
| **Deshmukh 2009** | 1 | 2 yr/F | AM | 11 | R lobe | Multi | Resection | Squamous | N | --- | NR |
| **Goodman 2009** | 1 | 55 yr/M | Incidental | 2 | GB fundus | Uni | Resection | N | N | --- | NR |
| **Oida 2009** | 1 | 57 yr/F | NR | 4.4 | S4 | Uni | Resection | N | ^B^FNA | --- | WNL |
| **Zhang 2009** | 1 | 60 yr/F | RUQ fullness | 7 | S4 | Multi | Resection | SCC | N | Absent smooth muscle layer | WNL |
| **Betalli 2008** | 1 | 14 mo/F | Incidental | 3 | S4 | Uni | Resection | N | N | Prenatal Dx (20 wks) | NR |
| **Geramizadeh 2008** | 1 | 25 yr/M | AP | 2.7 | S8 | Uni | Resection | N | N | Mimicked hydatid cyst | NR |
| **Kiyochi 2008** | 1 | 69 yr/F | Jaundice | 2.5 | S4 | Uni | Resection | N | N | --- | ↑CA19-9 |
| **Lubrano 2008** | 1 | 58 yr/M | Incidental | 1.9 | S4 | NR | Resection | Gastric | N | HCV, renal transplant | NR |
| **Shaw 2008** | 1 | 50 yr/F | AP | 8 | S5 | NR | Resection | N | N | --- | WNL |
| **Kaplan 2007** | 1 | 68 yr/M | AP, N/V, BM | 3 | S4 | Uni | Observation | N | ^B^FNA, Bx | --- | ↑CA19-9 |
| **Young 2007** | 1 | 16 yr/F | AP, anorexia | 6 | R lobe, PH | Uni | Resection | N | ^B^PQ-Cath | --- | NR |
| **Ben Mena 2006** | 1 | 31 yr/F | AP | 6 | S4 | Uni | Resection | Squamous | N | --- | WNL |
| **De 2006** | 1 | 70 yr/F | AP | 3.5 | S4 | Uni | Observation | N | ^B^CT-FNA | --- | NR |
| **Kang 2006** | 1 | 56 yr/M | Incidental | 4.5 | S4 | Uni | Resection | N | N | --- | WNL |
| **Sato 2006** | 2 | 72 yr/M | Incidental | 1.1 | S4 | Uni | Autopsy | N | N | Absent smooth muscle layer | NR |
|  |  | 69 yr/M | AP | 2 | S4 | Uni | Resection | N | N |  | NR |
| **Straus 2006** | 1 | 63 yr/M | Incidental | 1.5 | S4a | NR | Resection | N | N | Concomitant RCC | NR |
| **Stringer 2006, Rogers 2007** | 1 | 9 mo/M | Incidental | 7 | S5, 8 | Uni | Resection | Squamous | PQ-Asp | Prenatal Dx (30 wks) | NR |
| **Fang 2005** | 1 | 30 yr/M | AP, Fa | 4 | S4 | Uni | Resection | N | N | --- | NR |
| **Kim 2005** | 1 | 3 yr/M | Incidental | 1 | R lobe | Webbed | Resection | N | N | Satellite cysts | NR |
| **Koletsa 2005** | 1 | 39 yr/F | AP, N | 6.4 | S5 | Multi | Resection | N | N | Communicated with GB & CD | WNL |
| **Rodriguez 2005** | 1 | 20 yr/F | AP | NR | GB fossa | NR | NR | N | N | --- | NR |
| **Cai 2004** | 1 | 30/M | AP | 6 | S4 | Uni | Resection | N | N | --- | WNL |
| **Jakowski 2004** | 1 | 42 yr/F | AP, N/V | 2.1 | S4b | Uni | Resection | N | N | --- | ↑CA19-9 |
| **Momin 2004** | 1 | 68 yr/F | Incidental | 3.8 | S4c | Uni | Resection | N | N | --- | NR |
| **del Poggio 2003** | 1 | 58 yr/F | Incidental | 2 | S4 | Uni | Observation | N | ^B^FNA | --- | NR |
| **Horii 2003** | 1 | 47 yr/F | NR | 3.5 | S4 | Uni | Resection | N | FNA | --- | WNL |
| **Bogner 2002** | 1 | 55 yr/M | Incidental | 2.5 | R lobe | Uni | Autopsy | N | N | --- | NR |
| **de Lajarte-Thirouard 2002** | 1 | 40 yr/F | AP, thoracic pain, back pain | 13 | S5 | Uni | Resection | SCC, gastric/antral | N | --- | NR |
| **Furlanetto 2002** | 1 | 21 yr/M | AP, weight loss | 10 | S5, 6 | Multi | Resection, chemotherapy | SCC | US-FNA | --- | WNL |
| **Hirata 2001** | 1 | 53 yr/M | Incidental | 4 | S4 | Uni | Resection | N | US-FNA | HCV | NR |
| **Chatelain 2000** | 7 | 45 yr/M | Incidental | 3 | S4 | NR | Resection | N | N | --- | NR |
|  |  | 39 yr/F | Regurgitation | 4 | S4 | NR | Resection | N | N | --- | NR |
|  |  | 62 yr/M | Incidental | 2.5 | S4 | NR | Resection | N | N | --- | NR |
|  |  | 75 yr/F | AP | 3 | GB wall | NR | Resection | N | N | --- | NR |
|  |  | 47 yr/F | AP | 1.5 | S4 | NR | Resection | N | N | --- | NR |
|  |  | 51 yr/M | Incidental | 1 | S4 | NR | Resection | N | N | --- | NR |
|  |  | 43 yr/F | AP | 1 | S4 | NR | Resection | N | N | --- | NR |
| **Vick 1999**  **(*AJSP*)** | 6 | 14 yr/F | Jaundice | 6 | NR | Multi | Resection | N | N | --- | NR |
|  |  | 45 yr/M | Incidental | 3 | S4 | Uni | Resection | N | N | --- | NR |
|  |  | 48 yr/M | Incidental | NR | R lobe | Uni | Resection | N | N | --- | NR |
|  |  | 59 yr/F | Incidental | 3 | NR | Uni | Resection | N | N | --- | NR |
|  |  | 60 yr/F | Incidental | 1.1 | NR | Uni | Resection | N | N | --- | NR |
|  |  | 61 yr/M | Incidental | 2.5 | R lobe | Uni | Autopsy | N | N | --- | NR |
| **Vick 1999**  **(*Archives*)** | 1 | 51 yr/M | Incidental | 12 | R lobe | Uni | Resection | SCC | N | --- | NR |
| **Harty 1998** | 1 | 17 yr/F | AP, Fa | 8 | R lobe | Uni | Resection | Squamous | N | --- | NR |
| **Wu 1998** | 1 | 44 yr/F | AP | 5 | S4 | Bi | Resection | N | N | --- | ↑CA19-9 |
| **Carnicer 1996** | 1 | 5 yr/F | Incidental | 2 | PH | Uni | Observation | N | ^B^US-FNA | --- | NR |
| **Hornstein 1996** | 3 | 69 yr/M | Incidental | 3.5 | Anterior | Uni | Observation | N | ^B^US-FNA | --- | NR |
|  |  | 53 yr/F | Incidental | 3 | NR | NR | Observation | N | ^B^CT-FNA | --- | NR |
|  |  | 38 yr/M | AP | 3.5 | R lobe | Uni | Resection | N | ^B^US-FNA | --- | NR |
| **Murakami 1996** | 1 | 63 yr/F | Incidental | 2.2 | Medial | Uni | Resection | N | CT-Bx | Background cirrhosis | ↑CA19-9, ↑CEA |
| **Shoenut 1994** | 2 | 76 yr/M | AP, Na | NR | S4 | NR | Observation | N | ^B^CT-Bx | --- | NR |
|  |  | 82 yr/F | AP | NR | S4 | NR | Observation | N | ^B^CT-Bx | --- | NR |
| **Zaman 1994** | 1 | 35 yr/F | Incidental | 3 | S4 | Uni | Observation | N | ^B^CT-FNA | --- | NR |
| **Terada 1991** | 1 | 72 yr/F | NR | NR | NR | NR | NR | N | N | --- | NR |
| **Kadoya 1990, Terada 1990, Terada 1991** | 3 | 59 yr/F | Incidental | 4 | S4 | Uni | Resection | N | N | HBV | NR |
|  |  | 69 yr/M | R flank pain | 2.5 | S4 | Uni | Resection | N | N | --- | NR |
|  |  | 41 yr/M | Incidental | 3 | S4 | Uni | Resection | N | N | --- | NR |
| **Kimura 1990** | 1 | 67 yr/M | Incidental | 3.5 | L lobe | Uni | Resection | N | N | --- | NR |
| **Wheeler 1984** | 1 | 69 yr/M | Incidental | 2.5 | R lobe | Uni | Resection | N | N | --- | NR |
| **Dardik 1964** | 1 | 69 yr/F | AP | 9 | R lobe | Uni | Resection | N | N | --- | NR |
| **Dockerty 1956** | 4 | NR | NR | NR | NR | NR | NR | N | N | --- | NR |

^A^Tumor marker serologies include CA19-9, CEA, and/or AFP.

^B^Diagnostic fine needle aspiration (FNA)/core biopsy procedure.

**Additional file 1 legend** AD = abdominal distension; AM = abdominal mass; AP = abdominal pain; Bi = bilocular; BM = loose stool; Bx = core biopsy; Catheter = catheter drainage; CD = cystic duct; CM = centimeter; CT = computed tomography; Cyto = cytology; Dx = diagnosis; F = female; Fa = fatigue/lethargy; FNA = fine needle aspiration; GB = gallbladder; HBV = hepatitis B virus; HCV = hepatitis C virus; L = left; LFT = liver function test; M = male; Mo = month(s); Multi = multilocular; N = no; Na = nausea; N/V = nausea/vomiting; NR = not reported; PH = porta hepatis; PQ-Asp = percutaneous aspiration; PQ-cath = percutaneous catheter drainage; PVE = portal vein embolization; R = right; S = segment (of liver); SCC = squamous cell carcinoma; TACE = transarterial chemoembolization; Uni = unilocular; US = ultrasound; WNL = within normal limits; Yr = year.
